# A novel HMGA1-CCNE2-YAP axis regulates breast cancer aggressiveness

**DOI:** 10.18632/oncotarget.4236

**Published:** 2015-05-22

**Authors:** Silvia Pegoraro, Gloria Ros, Yari Ciani, Riccardo Sgarra, Silvano Piazza, Guidalberto Manfioletti

**Affiliations:** ^1^ Dipartimento di Scienze della Vita, Università degli Studi di Trieste, Trieste, Italy; ^2^ Laboratorio Nazionale CIB (LNCIB), Area Science Park, Trieste, Italy

**Keywords:** cyclin E2, YAP/TAZ, hippo pathway, CDK inhibitors, oncogene/tumour suppressor

## Abstract

High Mobility Group A1 (HMGA1) is an architectural chromatin factor that promotes neoplastic transformation and progression. However, the mechanism by which HMGA1 exerts its oncogenic function is not fully understood. Here, we show that cyclin E2 (CCNE2) acts downstream of HMGA1 to regulate the motility and invasiveness of basal-like breast cancer cells by promoting the nuclear localization and activity of YAP, the downstream mediator of the Hippo pathway. Mechanistically, the activity of MST1/2 and LATS1/2, the core kinases of the Hippo pathway, are required for the HMGA1- and CCNE2-mediated regulation of YAP localization. In breast cancer patients, high levels of HMGA1 and CCNE2 expression are associated with the YAP/TAZ signature, supporting this connection. Moreover, we provide evidence that CDK inhibitors induce the translocation of YAP from the nucleus to the cytoplasm, resulting in a decrease in its activity. These findings reveal an association between HMGA1 and the Hippo pathway that is relevant to stem cell biology, tissue homeostasis, and cancer.

## INTRODUCTION

Breast cancer is a complex and multistep disease involving the accumulation of both molecular and morphological changes within a cell. A critical step in the clinical outcome of breast cancer is the metastatic spread of cancer cells [1]. Understanding the molecular basis of metastasis is crucial for elucidating the mechanisms underlying this disease and designing an appropriate treatment.

The High Mobility Group A1 (HMGA1) gene encodes two proteins, HMGA1a and HMGA1b, which are generated via alternative splicing. Several studies have reported that HMGA1 expression is elevated in a variety of human cancers, including breast cancer, and enhanced HMGA1 protein expression has been associated with cancer metastasis [2-7]. Moreover, several reports have demonstrated a causal role of HMGA1 in inducing a transformed phenotype in cultured cells and in forming aggressive tumors in transgenic mice [8-10]. The oncogenic activities of HMGA1 are essentially due to its ability to modulate chromatin structure by preferentially binding to AT-rich DNA regions and to form stereospecific, multiple complexes defined as “enhanceosomes” that regulate the expression of genes involved in tumor progression and metastasis [11-13].

Several results strongly support a specific role for CCNE2 in breast cancer. CCNE2 has been detected in various prognostic gene expression profiles that predict a shorter metastasis-free survival or relapse-free interval in breast cancer patients [14-16]. CCNE2 overexpression in breast cancer cells induces genomic instability but does not affect mitotic progression [17, 18]. Therefore, even though it appears that CCNE2 might play a role in cancer progression, its underlying molecular mechanism is unknown.

The Hippo signaling pathway is a novel growth control and tumor suppressor pathway consisting of a kinase cascade comprising MST1/2 and LATS1/2 kinases [19-23]. Activation of the Hippo pathway results in the inactivation of YAP/TAZ proto-oncogenes via LATS1/2-mediated direct phosphorylation [22, 24]. Whereas phosphorylated YAP is sequestered in the cytoplasm, dephosphorylated YAP accumulates in the nucleus and acts primarily via the TEAD family of transcription factors to promote cell proliferation and organ growth. In tumors, YAP and TAZ are involved in the epithelial-to-mesenchymal transition (EMT), cancer stem cell properties, enhanced cell proliferation and acquisition of metastatic potential [25].

In this study, we investigated the contribution of HMGA1 and CCNE2 to breast cancer cell migration and invasion. Using basal-like breast cancer cell lines, we demonstrated that CCNE2 is an important downstream factor of HMGA1 that mediates tumor aggressiveness. We found that HMGA1 and CCNE2 exert their effect on cell migration by regulating YAP cellular localization and activity, through the action on the Hippo core kinases. Intriguingly, we found a clinically relevant relationship between HMGA1 and CCNE2 expression and the YAP/TAZ signature in breast cancer patients. Moreover, we show that CDK inhibitors effectively modulate YAP activity. Thus, this study identifies a novel HMGA1-CCNE2-YAP axis that regulates the metastasis of basal-like breast cancer, suggesting that this pathway might serve as a potential target for cancer therapy.

## RESULTS

### HMGA1 regulates CCNE2 in breast cancer cell lines

We recently identified a 130 gene-signature that is regulated by HMGA1 in breast cancer. This gene-signature displays prognostic value for breast cancer and correlates with a more aggressive and highly invasive basal-like cancer subtype [5].

To gain insight into the correlation between the expression level of these genes and the clinical data concerning poor patient prognosis, we constructed corresponding Cox proportional hazards models using public datasets of breast cancer gene expression. CCNE2 was among the genes that most highly correlated with poor clinical outcome and whose expression was tightly regulated by HMGA1 (Supplementary Figure S1a).

**Figure 1:**
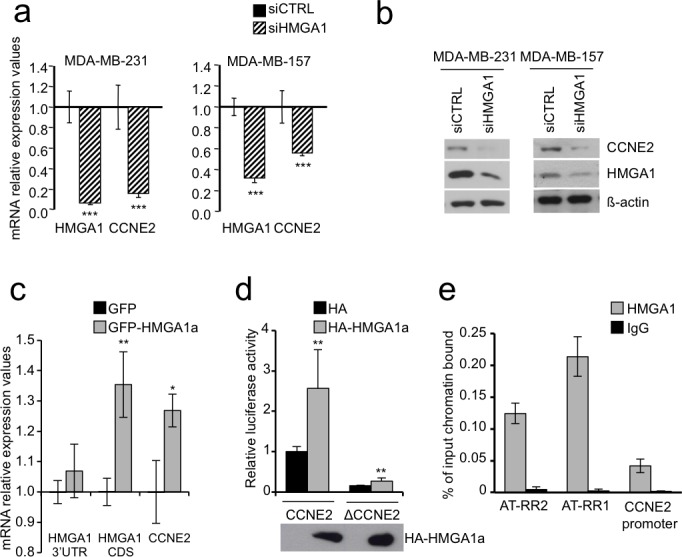
HMGA1 regulates CCNE2 expression **a.** CCNE2 mRNA expression was measured after 72 h of HMGA1 silencing (siHMGA1) in MDA-MB-231 and MDA-MB-157 cells using real-time RT-PCR. The level of CCNE2 expression in HMGA1 silenced cells was compared to that in cells transfected with control siRNA (siCTRL). GAPDH was used for normalization. The data are represented as the mean±SD (*n* = 3). See Supplementary Figure S1b for mRNA down-regulation of CCNE2 after HMGA1 silencing using siA1_1, which targets a different region of HMGA1 (*n* = 3). **b.** CCNE2 protein expression was analyzed after 72 h of HMGA1 silencing (siHMGA1) in MDA-MB-231 and MDA-MB-157 cells. A representative Western blot is shown. ß-actin was used as a loading control. See also Supplementary Figure S1c for protein down-regulation of CCNE2 after HMGA1 silencing using siA1_1. **c.** HMGA1 3′UTR, CDS and CCNE2 mRNA expression was measured after 30 h of GFP-HMGA1a overexpression in MDA-MB-231. 3′UTR amplification detects only endogenous HMGA1 while CDS amplification detects both endogenous and overexpressed HMGA1 mRNA because pEGFP-N1 HMGA1a vector contains only the HMGA1a coding sequence without 3′UTR. Levels of each mRNA were compared to that in cells transfected with control vector (GFP). GAPDH was used for normalization. The data are represented as the mean±SD (*n* = 3). **d.** HEK293 cells were transiently cotransfected with the luciferase reporter plasmid CCNE2 or ΔCCNE2 in combination with the expression plasmid pcDNA3HA or pcDNA3HA-HMGA1a. pRL-CMV Renilla luciferase expression vector was included to normalize for transfection efficiencies. Values are reported as relative luciferase activity comparing to cells transfected with the reporter vector CCNE2 and pcDNA3HA. The data are represented as the mean±SD (*n* > 5). Below the graph, Western blot of HA-HMGA1a. See Supplementary Figure S1d for luciferase assay on HEK293 silenced for HMGA1 and transfected with luciferase reporter plasmid CCNE2. **e.** Chromatin immunoprecipitation analysis of HMGA1 binding to CCNE2 promoter from MDA-MB-231 cells. Chromatin was immunoprecipitated with anti-HMGA1 antibody or rabbit purified IgG as negative control. Promoter occupancy was analyzed by real-time PCR amplifying three different region: AT-RR2 (from −8606 to −8469), AT-RR1 (from −2833 to −2714) and CCNE2 promoter (from −368 to −246) and calculated as percentage of input chromatin bound (*n* = 3). *P < 0.05, **P < 0.001***P < 0.001; two-tailed Student's *t*-test.

Next, we used basal-like breast cancer cell lines (MDA-MB-231 and MDA-MB-157) to assess whether CCNE2 expression is dependent on HMGA1 expression. Silencing of HMGA1 using a specific siRNA caused significant down-regulation of CCNE2 expression at both the mRNA and protein levels in the MDA-MB-231 and MDA-MB-157 breast cancer cell lines (Figure 1a and 1b). This effect was confirmed in MDA-MB-231 cells using a second independent siRNA against HMGA1 (Supplementary Figure S1b and c). At the same time, the overexpression of a tagged form of HMGA1a in MDA-MB-231 cells was able to up-regulate the expression of endogenous CCNE2 mRNA (Figure 1c).

We then tested whether the CCNE2 gene could be directly regulated by HMGA1 overexpressing a tagged form of HMGA1a together with a luciferase reporter gene construct containing the promoter region of CCNE2 (from – 580 to + 226). The overexpression of HMGA1a was able to increase the activity of the promoter while a deletion mutant ΔCCNE2 (+1 to + 223) had a much lower basal activity and it was weakly induced by HMGA1a (Figure 1d). Consistently, the silencing of endogenous HMGA1 expression was able to decrease the promoter activity of the reporter gene (Supplementary Figure S1d).

To further investigate whether CCNE2 is a direct target of HMGA1 *in vivo*, we performed chromatin immunoprecipitation (ChIP) assays in MDA-MB-231 cells. In addition of testing the promoter region we assayed also two more upstream regions, because from an inspection to the 5′ flanking region of the CCNE2 gene we observed that at −1.8 kb and at −8.6 kb there are two long regions of 3.0 kb and of 2.0 kb, that we named AT-rich region 1 (AT-RR1) and AT-rich region 2 (AT-RR2), with an unusual AT-rich composition, 62% and 55% respectively. A MatInspector analysis indicated the presence of several HMGA1 binding sites in these two regions and, in addition, the MAR-Wiz tool indicated in the more distant one (AT-RR2) a Scaffold/Matrix-Attachment-Region (S/MAR) suggesting the implication of these two putative regulatory elements in the regulation of the CCNE2 gene (Supplementary Figure S1e). ChIP experiments indicate that HMGA1 binds to the promoter region and, intriguingly, even more to AT-RR1 and AT-RR2 (Figure 1e), suggesting that HMGA1 could act through these two regulatory regions as well. Altogether therefore these results demonstrate that HMGA1 regulates CCNE2 expression by binding to the CCNE2 gene.

### HMGA1 expression is associated with that of CCNE2 in breast cancer patients

**Figure 2:**
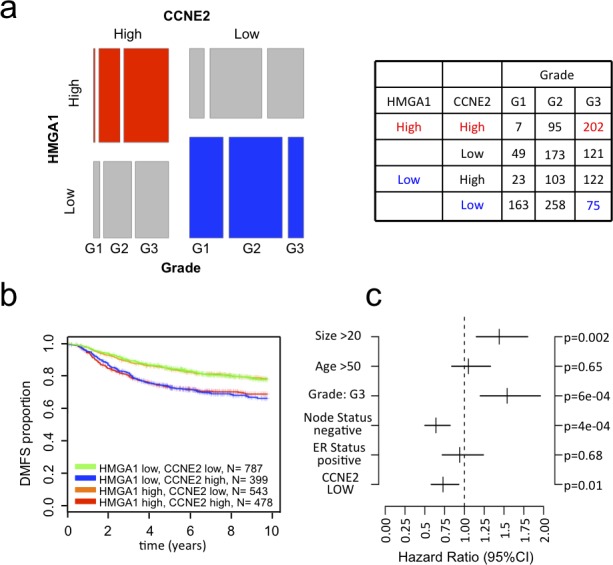
CCNE2 expression correlates with HMGA1 expression in breast cancer and tumor aggressiveness **a.** Mosaic plot showing the expression of HMGA1 and CCNE2 in breast cancer samples. The samples were stratified based on tumor grade and the expression of these two genes (higher or lower that the mean expression of the given gene in the meta-dataset). The table shows the number of samples for each category. **b.** Kaplan–Meier survival curves showing the relevance of CCNE2 and HMGA1 expression to clinical outcome, specifically DMFS. The patients were stratified based on the expression of these two genes. **c.** Multivariate analysis of CCNE2 expression in a breast cancer meta-dataset.

Next, we investigated the possible relationship between HMGA1 and CCNE2 expression in breast cancer patients. Based on the analysis of a breast cancer meta-dataset, we detected a strong correlation between the HMGA1 and CCNE2 expression levels (linear regression model, *P* < 10^−15^) (Supplementary Figure S2a). Then, we stratified breast cancer samples according to their relative expression levels of HMGA1 and CCNE2, obtaining a significant difference in patient distribution (Supplementary Figure S2b upper panel, chi-square, *P* < 10^−15^). A similar result was obtained using the TCGA breast cancer dataset (Supplementary Figure S2b lower panel, chi-square, *P* < 10^−8^). Moreover, when we classified patients according to grade and molecular subtype, we found concurrently elevated expression of both CCNE2 and HMGA1 in Grade 3 breast cancer and in the more aggressive breast cancer subtypes (luminal B and basal-like; Figure 2a and Supplementary Figure S2c). Interestingly, Kaplan-Meier survival analysis revealed that patients expressing high levels of both genes exhibited a significantly higher probability (*P* < 10^−15^) of developing distant metastasis than patients expressing low levels of both genes or patients in which only HMGA1 was highly expressed (Figure 2b). Furthermore, a high expression level of CCNE2 alone correlated with a poorer prognosis when HMGA1 expression levels were low. This finding suggests that CCNE2 serves as the effector molecule in the HMGA1-CCNE2 axis and that CCNE2 participates in conferring an aggressive phenotype to the tumor. This finding may also imply that CCNE2 expression, as expected, may be regulated by other transcriptional regulators because there is a subset of patients who express high CCNE2 levels and low HMGA1 levels. Taken together, these data suggest that the HMGA1-CCNE2 axis may mediate the oncogenic properties of breast cancer subtypes that are more undifferentiated and confer a poor prognosis. Kaplan-Meier survival analysis revealed that the expression of CCNE2 alone significantly correlated with clinical outcome in breast cancer patients (Supplementary Figure S2d). Moreover, a multivariate analysis of a cohort of 1131 breast cancer patients (a subset of our meta-dataset) revealed that a low expression level of CCNE2, as well as negative lymph node status, is a significant (*P* = 0.01) independent factor of good prognosis (Figure 2c).

### CCNE2 acts downstream of HMGA1 to regulate the motility of breast cancer cells

**Figure 3:**
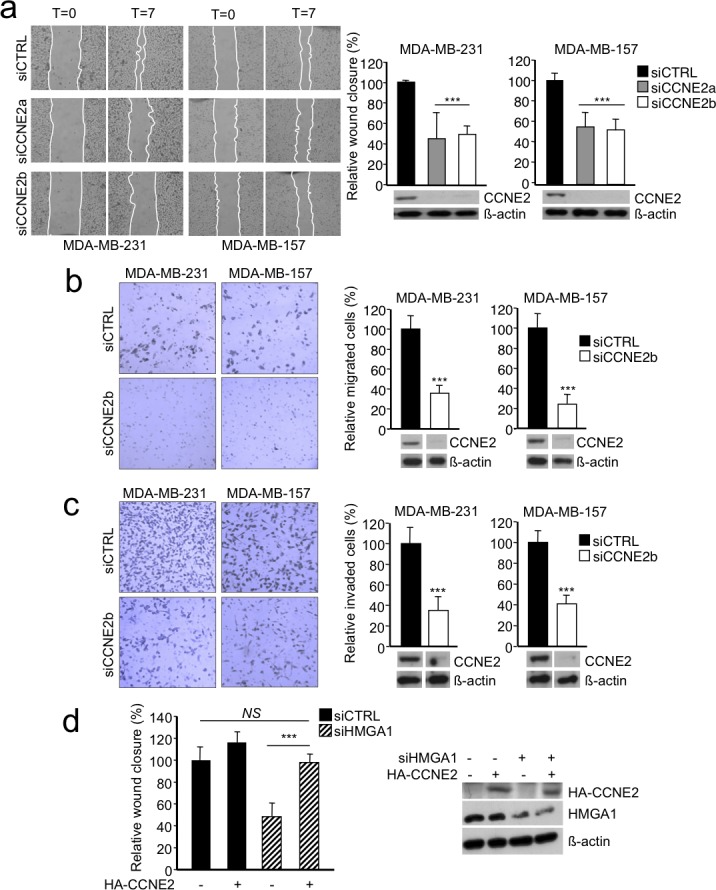
CCNE2 silencing impaired the migration and invasion of basal-like breast cancer cells **a.** Left, representative images of a wound-healing assay in which MDA-MB-231 and MDA-MB-157 cells were transfected with control siRNA (siCTRL) or two different siRNAs against CCNE2 mRNA (siCCNE2a or siCCNE2b) are shown. Confluent cell cultures were scratched, and wound closure was analyzed after 7 h with respect to time zero. Right, quantification of the wound-healing assay is presented as the means of the percentage of wound closure relative to the control±SD (*n* > 3). A representative Western blot of CCNE2 silencing in each cell line is shown. ß-actin was used as a loading control. **b.** Left, a representative transwell migration assay performed on MDA-MB-231 and MDA-MB-157 cells transfected with siCTRL or siCCNE2b is shown. Right, quantification of the transwell migration assay. **c.** The transwell invasion assay using the same cell lines used in b. Left, representative images of cells that migrated across the porous membrane that were stained with crystal violet. Right, quantification of the transwell assay. Below, a representative Western blot of CCNE2 silencing is shown. ß-actin was used as loading control. The data in b and c are presented as the mean of the percentage of the number of cells relative to the control±SD (*n* > 3). See Supplementary Figure S3 for transwell experiments using siCCNE2a. **d.** Analysis of a wound-healing assay in MDA-MB-231 cells co-transfected with siCTRL or siHMGA1 and a vector expressing HA-CCNE2. The empty vector (−) was used as a negative control. The data are presented as the means of the percentage of wound closure relative to the control±SD (*n* = 4). Right, a representative Western blot analysis of the cell lysates is presented. ß-actin was used as a loading control. ***P < 0.001; two-tailed Student's *t*-test.

In breast cancer cells, HMGA1 is primarily involved in regulating cell motility and invasiveness [5, 6]; however, its downstream effectors are not well characterized. Therefore, we explored the functional relevance of CCNE2 in cell motility and invasiveness. Initially, we performed a wound-healing assay after CCNE2 silencing. Down-regulation of CCNE2 using two different siRNAs significantly reduced the 2D migration ability of both MDA-MB-231 and MDA-MB-157 cells (Figure 3a). To specifically analyze the migration component of CCNE2 activity, we performed Boyden chamber assays and found that cell migration was significantly impaired in MDA-MB-231 and MDA-MB-157 cells (Figure 3b and Supplementary Figure S3a). Moreover, using Matrigel-coated inserts, we clearly determined that the invasion properties of all cell lines were dramatically decreased by CCNE2 silencing (Figure 3c and Supplementary Figure S3b), suggesting a direct involvement of CCNE2 in metastasis.

The data described above establish CCNE2 as a participant in breast cancer cell migration and invasion. Next, we sought to verify whether CCNE2 serves as a downstream effector of HMGA1-induced cell migration. Indeed, the reintroduction of CCNE2 expression using pcDNA3HA-CCNE2 rescued the migration of HMGA1-silenced MDA-MB-231 cells to levels comparable to those of control cells (Figure 3d).

Next, we examined whether silencing of CCNE2 affects breast cancer cell proliferation. CCNE2 silencing did not significantly alter the proliferation of MDA-MB-231 or MDA-MB-157 cells (Supplementary Figure S4a). Moreover, cell cycle analysis revealed a slight accumulation of cells in the G0/G1 phase only in the MDA-MB-157 cell line (Supplementary Figure S4b).

Taken together, these results suggest that CCNE2 plays a specific role in promoting cell migration and invasion in metastatic breast cancer cell lines downstream of HMGA1.

### HMGA1 and CCNE2 regulate the nuclear localization and activity of YAP

**Figure 4:**
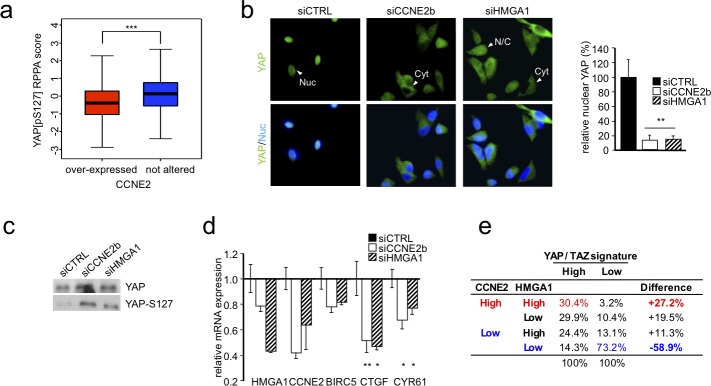
HMGA1 and CCNE2 affect YAP localization and activity **a.** The boxplot shows the YAP protein phosphorylation level in breast cancer samples. The samples were stratified based on the expression level of CCNE2. **b.** Representative immunofluorescence images of YAP in MDA-MB-231 cells after HMGA1 and CCNE2 silencing are shown. Representative cells are indicated by arrowheads with abbreviations (Nuc, primarily nuclear; Cyt, primarily cytoplasmic; N/C, diffuse in the nucleus and the cytoplasm). Images were taken at X 60 magnification. Right, the ratio of percentage of cells with nuclear YAP treated with siCCNE2b and siHMGA1 compared with siCTRL. The data are presented as the mean±SD (*n* = 3). **c.** Western blot analysis of total YAP and YAP phosphorylated at Ser127 (YAP-S127) in protein lysates of MDA-MB-231 cells transfected with siCTRL or siHMGA1 and siCCNE2b (*n* ≥ 5). **d.** Real time RT-PCR analysis of YAP target genes (CTGF, BIRC5, CYR61) in MDA-MB-231 cells after HMGA1 and CCNE2 silencing. GAPDH was used for normalization. The data are presented as the mean±SEM (*n* = 4). **e.** Contingency table frequencies of breast cancer samples (TCGA dataset) classified as expressing high or low levels of YAP/TAZ profile genes or displaying high or low HMGA1 and CCNE2 expression levels. *P < 0.05, **P < 0.01, ***P < 0.001; two-tailed Student's *t*-test.

Next, we examined the mechanism by which CCNE2 mediates cell migration and invasion. To address this issue, we explored whether alterations in CCNE2 expression correlate with RNA levels and protein phosphorylation levels by analyzing a cohort of 408 breast cancer patient samples via RNA-seq and phospho-proteomics approaches. Specifically, we categorized patients according to their CCNE2 expression levels and ranked the differential changes obtained from reverse phase protein array (RPPA) analysis. Among the most significant differential phosphorylations, we found an inverse relationship between CCNE2 expression and the phosphorylation of YAP at Ser127 (Figure 4a). Phosphorylation of YAP at Ser127 induces YAP translocation from the nucleus to the cytoplasm, repressing its activity [22].

Thus, we hypothesized that HMGA1 and CCNE2 participate in breast cancer cell migration via YAP by interfering with YAP phosphorylation at Ser127 and promoting its nuclear localization. Therefore, we silenced HMGA1 and CCNE2 in MDA-MB-231 and MDA-MB-157 cells and we evaluated YAP localization via immunofluorescence analysis. As shown in Figure 4b and Supplementary Figure S5a and S5b, in the HMGA1- and CCNE2-silenced cells, YAP predominantly displayed a cytoplasmic cell distribution, losing its nuclear localization.

To further support our hypothesis, the phosphorylation status of YAP at Ser127 was measured using a specific antibody in MDA-MB-231 cells in which CCNE2 and HMGA1 were silenced. As shown in Figure 4c, silencing both HMGA1 and CCNE2 in MDA-MB-231 cells increased the phosphorylation levels of YAP at Ser127. This result suggests that this modification may be responsible for the observed cytoplasmic relocalization of YAP and confirms in an *in vitro* model the inverse correlation between CCNE2 expression and YAP Ser127 phosphorylation that was found in the tumor samples.

Furthermore, we monitored the effect of CCNE2 and HMGA1 knockdown on well-established endogenous YAP target genes, such as BIRC5, CYR61 and CTGF. We found that CTGF and CYR61 were significantly down-regulated upon HMGA1 and CCNE2 silencing, whereas BIRC5 appeared to be down-regulated, although this result was not significant (Figure 4d). Therefore, these results demonstrate that both CCNE2 and HMGA1 regulate the YAP target gene expression pattern. This important association was confirmed in breast cancer patients (TCGA breast cancer dataset). In fact, the differential activation of the YAP/TAZ signature is associated with the expression levels of both the CCNE2 and HMGA1 genes (Figure 4e, Chi-square, *P* < 10^−15^).

### HMGA1 and CCNE2 regulate YAP through MST1/2 and LATS1/2 kinases

**Figure 5:**
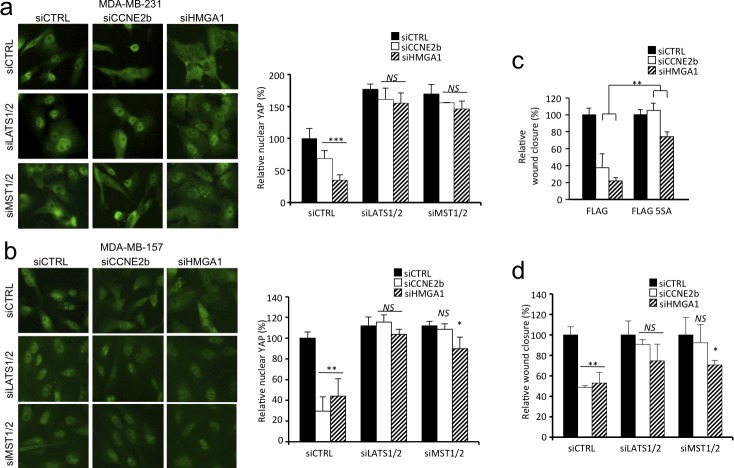
HMGA1 and CCNE2 regulate YAP localization in a MST1/2 and LATS1/2 kinase-dependent manner in breast cancer cells **a.** Immunofluorescence images of YAP in MDA-MB-231 cells, indicating the effect of LATS1/2 and MST1/2 silencing on cells in which HMGA1 and CCNE2 were depleted. In the graph, the ratio of nuclear YAP relative to siCTRL±SD is presented (*n* = 3). Images were taken at X 40 magnification. **b.** The same experiment presented in a. performed in MDA-MB-157 cells. **c.** Wound-healing assay using MDA-MB-231 cells stably infected with pBABE-FLAG (FLAG) or pBABE-FLAG-YAP-5SA (FLAG-5SA) after transfection with siCTRL, siCCNE2b or siHMGA1 for 72 h. The data are presented as the means of the percentage of wound closure relative to each control (siCTRL)±SD (*n* = 3). See Supplementary Figure S5c for Western blot analysis. **d.** Wound-healing assay using MDA-MB-231 cells treated as in panel a. *P < 0.05, **P < 0.01, ***P < 0.001, *NS* = not significant; two-tailed Student's *t*-test.

MST1/2 and LATS1/2 constitute the Hippo core kinase cassette and are the primary kinases responsible for YAP/TAZ phosphorylation and inactivation [22, 24], we therefore asked whether these kinases mediate the effect of HMGA1 and CCNE2 on YAP nuclear localization. Silencing of LATS1/2 and MST1/2 in MDA-MB-231 cells almost completely rescued the effect of HMGA1 and CCNE2 depletion on YAP nuclear localization (Figure 5a). The rescue on nuclear localization following LATS1/2 and MST1/2 silencing was also confirmed in another cell line (MDA-MB-157) (Figure 5b).

To address whether YAP serves as a mediator of HMGA1- and CCNE2-induced cell migration, we used a constitutively active form of YAP carrying serine-to-alanine substitutions at the primary YAP phosphorylation sites (YAP-5SA) [22], including the LATS1/2-targeted sites Ser127 and Ser381. The constitutive expression of YAP-5SA was sufficient to counteract the effect of HMGA1 and CCNE2 silencing on MDA-MB-231 cell migration (Figure 5c and Supplementary Figure S5c).

We then assessed whether LATS1/2 and MST1/2 kinases were responsible for the YAP-mediated HMGA1 and CCNE2 effect on cell migration. Silencing of LATS1/2 and MST1/2 rescued almost completely the effect of HMGA1 and CCNE2 depletion on cell motility (Figure 5d). These results therefore demonstrate that the activity of HMGA1 and CCNE2 is largely dependent on the core kinases of the Hippo pathway.

### CDK inhibitors regulate the nuclear localization of YAP and cell migration

**Figure 6:**
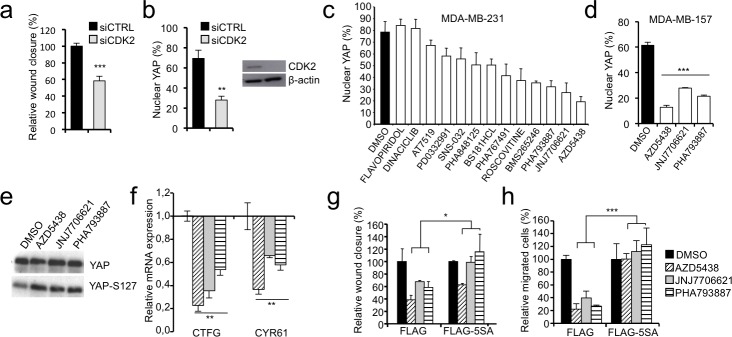
CDKs affect YAP localization and the motility of breast cancer cells **a.** Wound-healing assay in MDA-MB-231 cells transfected with control siRNA (siCTRL) or a pool of siRNAs against CDK2, data is presented as the means of the percentage of wound closure relative to the control±SD (*n* > 3). **b.** Immunofluorescence analysis of YAP localization in MDA-MB-231 treated as in a. Data is presented as percentage of cells with nuclear YAP in each condition. **c.** Results of the CDK inhibitor library screening evaluating YAP localization. MDA-MB-231 cells were treated for 24 h with 1 mM of inhibitor, except for SNS-032 and DINACICLIB, which were applied at 0.1 mM. The data are presented as the means of percentage of cells with nuclear YAP±range between replicates (*n* = 2). See Supplementary Figure S6a for representative images. **d.** Immunofluorescence analysis of YAP localization in MDA-MB-157 treated with the most effective CDK inhibitors (AZD5438, JNJ7706621 or PHA793887). Data is presented as percentage of cells with nuclear YAP in each condition (*n* = 3). See Supplementary Figure S6a for representative images. **e.** Western blot of total YAP and phosphorylated YAP at Ser127 (YAP-S127) in protein lysates of MDA-MB-231 cells treated with CDK inhibitors (*n* = 3). **f.** Real time RT-PCR analysis of YAP target genes (CTGF and CYR61) in MDA-MB-231 cells treated with CDK inhibitors. GAPDH was used for normalization. The data are presented as the mean±SD (*n* = 3). **g**. and **h**. Wound-healing assay (g) and transwell migration assay (h) using MDA-MB-231 cells stably infected with pBABE-FLAG (FLAG) or pBABE-FLAG-YAP-5SA (FLAG-5SA) after the treatment with CDK inhibitors. See Supplementary Figure S7a and b for representative images of wound-healing and transwell respectively. Experiments presented in panel d-h were performed in cells treated with AZD5438, JNJ7706621 or PHA793887. The data are presented as the means of the percentage relative to each control (DMSO)±SD (*n* = 3). *P < 0.05, **P < 0.01, ***P < 0.001; two-tailed Student's *t*-test.

We demonstrated that CCNE2 and HMGA1 intercept the Hippo pathway by regulating YAP phosphorylation and nuclear localization. Because the functions of CCNE2 are predominantly dependent on CDK2 [26], we evaluated whether CDK2 depletion altered YAP localization and cell motility. Figure 6a and 6b show that following CDK2 silencing both cell motility and YAP nuclear localization are severely impaired. Thus, to test whether CDK inhibitors could be exploited as potential inhibitors of YAP activity, we performed a screen using a panel of CDK inhibitors and assessed YAP localization in MDA-MB-231 cells. Strikingly, most of the tested compounds affected YAP nuclear localization (Figure 6c and Supplementary Figure S6a). The three most effective inhibitors (AZD5438, JNJ7706621 and PHA793887) were also tested in another breast cancer cell line, MDA-MB-157, obtaining similar results (Figure 6d and Supplementary Figure S6a). Then, we analyzed the phosphorylation status of YAP at Ser127 after treatment of MDA-MB-231 with the three most effective compounds and found an increase in YAP phosphorylation at Ser127 concomitant with the cytoplasmic translocation of YAP (Figure 6e). Consistently all three inhibitors were able to down regulate the expression of the two YAP target genes CTFG and CYR61 that we showed to be affected by HMGA1 and CCNE2 silencing (Figure 6f). Next, we examined whether CDK inhibitors alter the migratory capacity of MDA-MB-231 cells and whether this function is associated with the cytoplasmic translocalization of YAP. All three inhibitors significantly impaired cell migration without appreciably affecting cell proliferation at the concentration and time points evaluated (Figure 6g and 6h and Supplementary Figure S6b). Notably, this effect was specifically mediated by YAP; in fact, the expression of the mutant YAP-5SA rescued cell migration, albeit at different levels for the three inhibitors (Figure 6g and 6h and Supplementary Figure S7a and b).

## DISCUSSION

We recently showed that HMGA1 promotes metastatic processes in basal-like breast cancer cells by regulating the EMT and stemness via the activation of a specific gene signature [5]. Among those genes, we found that CCNE2 acts downstream of HMGA1 to mediate breast cancer metastasis. A growing body of evidence indicates that CCNE2 is involved in breast cancer cell migration [27-29]. Indeed, CCNE2 is a component of three prognostic gene expression signatures that predict shorter metastasis-free survival [14-16]. Here, we show that depletion of CCNE2 strongly impairs the migration and invasion of breast cancer cells without significantly altering their proliferation. In recent years, it has become evident that cyclins and cyclin-dependent proteins play cell cycle-independent roles [27, 30-32]. Cyclin D1 promotes cell migration by regulating the RhoA-ROCK pathway [33], that can in turn regulate the Hippo pathway [34-36]. Intriguingly, cyclin D1 is regulated by HMGA1 [37]. Therefore, our study extends the current understanding of the alternative roles of cyclins in cell migration, identifying CCNE2 as a novel mediator of the migration of breast cancer cells and suggesting a wider role of cyclins in regulating the Hippo pathway.

HMGA1 is widely involved in tumorigenesis due to its ability to interact with transcription factors and to modulate chromatin plasticity. Via these properties, HMGA1 is considered a key hub for several oncogenic pathways, such as the Wnt/ß-catenin and Notch pathways [5, 38] and Ras/ERK signaling [39]. In this study, we provide the first evidence that HMGA1 regulates the Hippo pathway via CCNE2, which ultimately modulates the activity of YAP. We demonstrate that HMGA1 is able to bind *in vivo* to CCNE2 promoter and activate CCNE2 transcription. The promoter region of CCNE2 was shown to contain *in vivo* binding sites for and to be activated by E2F1 [40, 41]. Because it was demonstrated that HMGA1, by binding to Rb, was able to activate E2F target genes [42, 43] it is likely that this mechanism could be involved in the regulation of CCNE2 transcription as well. In addition, we provide evidence that HMGA1 can bind to two AT-rich regions, AT-RR1 and AT-RR2, upstream the CCNE2 promoter, one of which, AT-RR2, is a potential S/MAR. HMGA1 is a nuclear architectural factor that can organize local chromatin structures regulating gene expression by binding to long AT-rich stretch and to S/MAR (4, 44, 45). Therefore it is possible that AT-RR1 and AT-RR2 distal elements could regulate CCNE2 transcription by physically interacting with the CCNE2 promoter through HMGA1-mediated chromatin looping.

We found that inhibition of HMGA1 and CCNE2 induced YAP inactivation by promoting its cytoplasmic localization, and we showed that this effect is mediated by MST1/2 and LATS1/2, the core kinases of the Hippo pathway. To examine the relevance of the HMGA1- and CCNE2-mediated inhibition of YAP to the induction of cell migration, we used a constitutively active form of YAP (YAP-5SA). This mutant rescued the effect of HMGA1 and CCNE2 silencing on cell migration. These results suggest that the HMGA1-CCNE2-YAP axis acts via the Hippo pathway to modulate oncogenic properties, such as cell migration. The mechanism by which HMGA1 modulates the activity of MST1/2 and LATS1/2 remains an open question that merits further investigation.

These results might also be relevant to developmental studies. Several reports have indicated that HMGA1 and the highly related HMGA2 protein are implicated in regulating body size. Indeed, knockout of *Hmga2* is responsible for the *pygmy* phenotype in mice [46], and *Hmga1* and *Hmga2* double knockout results in a super *pygmy* phenotype [43]. The Hippo pathway constitutes an intrinsic regulator of organ size, as well. Inactivation of this cascade causes excess nuclear accumulation of YAP/TAZ, leading to organ overgrowth [21, 47, 48]. Thus, we propose that these two pathways might cooperate during development, as well as during cancer.

The Hippo signaling pathway is frequently deregulated in many different cancers, including breast cancer [25]. Therefore, pharmacological inhibition of YAP/TAZ activity may represent an effective anticancer strategy. However, to date, few small molecule inhibitors that target the Hippo pathway have been discovered. Among these inhibitors, statins have very recently been shown to display the potential to target the malignant effects of YAP/TAZ in cancer cells [49, 50]. Because inhibition of a single CDK can be overcome by the compensatory activity of other CDKs [51], we evaluated the effects of CDK pan-inhibitors on YAP localization and showed that several CDK inhibitors effectively inhibit YAP, decreasing its nuclear localization and activity impairing cell migration. CDK inhibitors are considered attractive compounds for blocking CDK activities, primarily consisting of regulation of the cell cycle and cell proliferation [51]. An intensive search is on-going for possible therapeutic applications of CDK inhibitors; in fact, several CDK inhibitors are under investigation in clinical trials. Interestingly, their potential to inhibit tumor growth in mice has been demonstrated [52]. Moreover, we found that CDK inhibitors decrease the migratory capacity of breast cancer cells. Therefore, we suggest investigating the efficacy of these compounds for their ability to reduce metastasis *in vivo*.

In conclusion, we demonstrate that the novel HMGA1-CCNE2 axis regulates cell migration by intercepting the Hippo pathway and, ultimately, by modulating YAP activity. Moreover, we propose the use of CDK inhibitors to target the Hippo pathway.

## MATERIALS AND METHODS

### Cell culture and treatments

MDA-MB-231, MDA-MB-157 and HEK-293 cell lines were cultured in DMEM containing 10% tetracycline-free FBS, 2 mM L-glutamine, 100 U/ml penicillin and 100 μg/ml streptomycin. For transfection of siRNA, all cell lines were transfected with Lipofectamine^TM^ RNAiMAX reagent (Invitrogen) for 72 h. The siRNAs against HMGA1 (siA1_1 and siA1_3, referred to as siHMGA1 throughout the text) [5] and the siRNA against CCNE2 (siCCNEa) were used previously [53]. siCCNE2b -5′ GAAAGCCUCAGGUUUGGAG 3′- was designed using an Invitrogen tool to target exon 10; siLATS1/2 was used previously [49]; for MST1/2 silencing we used a pool of siRNAs composed by siMST1 -5′ GCAGGUCAACUUACAGAUA 3′- and siMST2 -5′ CCACAAGUACAAAGACCAU 3′-; for CDK2 silencing we used a pool of siRNAs composed by siCDK2_1 -5′ GCUUGGCCUUGGGCUAUUU 3′- and siCDK2_2 -5′ GCCUUCCUACACGUUAGAU 3′-. siMST1/2 and siCDK2s were designed using an Invitrogen tool. Plasmid transfections were performed using either FuGENE^HD^ (Promega) or Lipofectamine 3000 (Invitrogen) or standard Calcium Phosphate method. For pcDNA3HA-CCNE2 plasmid construction, the CCNE2 coding sequence was amplified from reverse-transcribed total RNA of MDA-MB-231 cells using the following primers: forward -5′ TTAACGGAATTCATGTCAAGACGAAGTAGC 3′- and reverse -5′ TCTCCTCGAGTTATTAGTGTTTTCCTGGTGG 3′- and the resulting fragment was cloned into *EcoR*I and *Xho*I restriction sites of pcDNA3HA. The plasmid DNA was checked by sequencing. For functional-rescue experiments, the cells were initially transfected with siRNA; 24 h later, the cells were transfected with plasmid pcDNA3HA or pcDNA3HA-CCNE2 using FuGENE^HD^ (Promega), experiments were done 48 h after. For overexpression experiment, MDA-MB-231 cells were transfected with plasmids pEGFP-N1 or pEGFP-N1 HMGA1a, that was a kind gift from Prof. G. Giannini, using Lipofectamine 3000 (Invitrogen), experiment was done 30 h after transfection. For experiments using YAP-5SA, MDA-MB-231 cells were initially transfected with pBABE-FLAG and pBABE-FLAG-YAP-5SA [49], selected using puromycin and then silenced for 72 h using siCTRL, siA1_3 or siCCNE2b. For LATS1/2 and MST1/2 silencing, the cells were initially transfected with siLATS1/2 or siMST1/2; after 24 h, the cells were transfected with siHMGA1 and siCCNE2b for 48 h. For CDK inhibitor screening, cells were treated with PD03329911, SNS-032, ROSCOVITINE, PHA793887, AT7519, BS181HCl, BMS265246, AZD5438, FLAVOPIRIDOL, PHA767491, PHA848125, DINACICLIB or JNJ7706621 for 24 h (Sellekchem). These inhibitors were applied at 1 μM, except for SNS-032 and DINACICLIB, which were applied at 0.1 μM.

### Immunoblotting

The cells were washed in chilled PBS and lysed using SDS sample buffer [62.5 mM Tris, pH 6.8; 2% SDS; 10% glycerol; 50 mM DTT; 1 mM Na_3_Vo_4_; 5 mM NaF; and mammalian protease inhibitor cocktail (PIC) (Sigma)]. The lysates were separated via SDS-PAGE prior to transfer to nitrocellulose membranes (GE-Healthcare). Western blot analyses were performed according to standard procedures using the following antibodies: anti-CCNE2 (Abcam); anti-ß-actin (Sigma); anti-HMGA1 [5]; anti-YAP (Santa Cruz); anti-P127-YAP (Novus Biological); and anti-FLAG (Sigma).

### Immunostaining

The cells were grown at low density on glass slides and fixed with 4% PFA. After permeabilization with 0.3% Triton/PBS and saturation in 0.5% BSA/PBS, the cells were incubated in the anti-YAP primary antibody (Santa Cruz) diluted in PBS containing 0.5% BSA and 0.01% Triton. The anti-mouse Alexa 488 secondary antibody (Invitrogen) was applied. Then, the cells were stained with Hoechst to detect the nuclei. The images were visualized using a Nikon Eclipse e800 microscope and acquired using Nikon ACT-1 software.

### Migration and invasion assays

For the wound-healing assays, the cells were cultured to confluence on 35-mm plates. Then, the cells were scraped using a 200-μl tip, and wound closure was monitored for 7 h. Images of the same area were captured for each plate, and wound closure was analyzed using ImageJ software. For the transwell migration and invasion assays, 24-well PET inserts were used (8.0-mm pore size, Falcon) without or with Matrigel-coated filters for invasion; 40,000 and 100,000 cells were seeded, respectively. The migrated and invaded cells were fixed after 22 h and 24 h, respectively, in 4% PFA and were stained with 0.5% crystal violet (Sigma).

### MTS cell growth analysis

First, 5,000 cells were seeded in 96-well dishes. Every 24 h, cell growth was measured via a CellTiter 96^®^ AQueous One Solution Cell Proliferation Assay (Promega) according to the manufacturer's instructions. At each time point, the medium in each well was replaced with a solution composed of 100 μl of PBS containing 4.5 g/L glucose (Sigma) and 20 μl of CellTiter 96^®^ AQueous One Solution.

### Promoter analysis

Putative MARs were mapped using MAR-Wiz (http://genomecluster.secs.oakland.edu/cgi-bin/mar-upload.cgi), with default parameters. Putative HMGA1 binding site were obtained using MatInspector tool (http://www.genomatix.de/cgi-bin//eldorado/main.pl). The analyzed sequence is from +226 to – 11430 of CCNE2 gene.

### Luciferase assay

HEK-293 cells were plated at density of 350,000 cells per 35-mm-diameter culture dish and processed 46.5 h after standard calcium phosphate transfection. Cells were transfected with 500 ng of the reporter construct, 1 μg of pcDNA3HA or pcDNA3HA-HMGA1a, and 50 ng of pRL-CMV Renilla luciferase expression vector (Promega) to normalize for transfection efficiencies. Reporter constructs are: pGL4-CCNE2 (kindly given by Jay A. Nelson laboratory) and pGL4-ΔCCNE2 that was obtained by amplifying a fragment of 223nt from pGL4-CCNE2 using the following primers: forward AATCTCGAGGTGCGGGGCGGGAC and reverse ACCCAAGCTTACGGAACGCGGGAACCCA and cloned in *Hind*III and *Xho*I restriction sites of pGL4.11 (Promega). The assays were performed with dual-luciferase reporter assay system (Promega) according to the manufacturer's instruction.

### Chromatin immunoprecipitation

MDA-MB-231 cells were cross-linked in culture medium with 1 % formaldehyde for 10 min, neutralized using 125 mM glycine in PBS for 5 min and washed in PBS. Nuclei were obtained by hypotonic buffer (5 mM Pipes pH 6.8, 85 mM KCl, 0.5 % NP-40 and protease inhibitors) and centrifugation. The nuclei pellet was resuspended in RIPA 100mM buffer (20 mM Tris-HCl pH 7.5, 100 mM NaCl, 1 mM EDTA, 0.5% NP-40, 0.5% Na-Deoxycholate, 0.1% SDS supplemented with protease inhibitors). Chromatin was sonicated to 500-800 bp average fragment size and precleared for 1 h at 4°C with protein A/G PLUS-Agarose (Santa Cruz Biotechnologies). Agarose was removed by centrifugation and an aliquot of supernatant was taken as input. Chromatin was immunoprecipitated overnight at 4°C with anti-HMGA1 polyclonal antibody. As a negative control for immunoprecipitation, IgGs purified from rabbit serum were used (Abcam). DNA protein complexes were recovered with protein A/G PLUSAgarose and washed sequentially with RIPA 100 mM buffer, RIPA 250 mM buffer (20 mM Tris-HCl pH 7.5, 250 mM NaCl, 1 mM EDTA, 0.5% NP-40, 0.5% Na-Deoxycholate and 0.1% SDS), LiCl solution (10 mM Tris-HCl pH 8.0, 1 mM EDTA, 250 mM LiCl, 0.5% NP-40 and 0.5% Na-Deoxycholate) and TE. To reverse cross-linking, samples were incubataed overnight at 65°C and after that RNase A (35 ng/μl) and proteinase K (90ng/μl) treatment was performed for 2 h at 55°C (0.2%, SDS, 35mM Tris HCl pH6.5, 8.8 mM EDTA pH 8). In parallel, input was treated in the same way. After phenol/chloroform extraction and ethanol precipitation samples were resuspended in H_2_O. Coimmunoprecipitated DNA was analyzed by Real Time PCR. Promoter occupancy was calculated as percent of chromatin input immunoprecipitated using the 2^−ΔCt^ method. Primer sequences are available on request.

### Gene expression analysis

Total RNA was extracted using TRIzol reagent (Invitrogen), subjected to DNase-I (Invitrogen) treatment and subsequently purified using phenol-chloroform. For quantitative RT-PCR, mRNA was transcribed using Superscript III (Invitrogen). Quantitative PCR was performed using SYBR Green PCR master mix (Applied Biosystems) and a 7500 Real-Time PCR System (Applied Biosystems). The primer sequences used are available upon request.

### Analysis of DNA/protein content (PI/FITC staining)

For each sample, the cells (10^6^) were fixed in 70% ethanol, pre-hydrated in PBS for 6 minutes, washed twice with PBS, and allowed to balance in PBS for 1 h. The cells were stained overnight with 500 μl of a PBS-based solution containing 10 μg of PI and 0.25 ng of FITC (all from Sigma). All flow cytometric measurements were performed using a CYTOMICS^TM^ FC500 (Beckman Coulter Inc. Fullerton, CA, USA) equipped with an Argon laser (488 nm, 5 mV) and configured according to standard parameters for green-filtered (525 nm, FL1) and red-filtered (610 nm, FL3) fluorescent detection (http://dsv.units.it/Dipartimento/fcs_dsv). After acquisition of at least 10,000 events per run, the data, which were stored as list mode files, were analyzed using FCS Express V3 software; alternatively, the saved FL3 histograms were subjected to cell cycle analysis, which was performed using MultiCycle^®^ software.

### Breast cancer gene expression data

Data processing was performed using several BioConductor packages (survival, affy and limma; see http://www.bioconductor.org/) in the R Computing Environment version 3.0.2 (http://www.r-project.org/).

#### Breast cancer meta-dataset

Several published gene expression datasets (breast cancer meta-dataset) were collected from the gene expression omnibus (GEO) public gene expression database (GSE1456, GSE4922, GSE5327, GSE6532, GSE7390, GSE11121, GSE12093, GSE2603, GSE16446, GSE19615, GSE20685 and GSE21653). The data were normalized in the R/Bioconductor environment using the RMA normalization method (affy package), generating a breast cancer meta-dataset. Gene annotation was obtained from brainarray custom CDF metadata packages, and the probe sets were converted to Entrez Gene Id and Symbol Id. Each dataset was analyzed separately to avoid platform and signal merging issues, and only the results were combined.

#### Breast cancer TCGA dataset

Gene expression data for the breast cancer samples (TCGA data set) were obtained from the Memorial Sloan Kettering Cancer Genomics Portal (http://www.cbioportal.org/public-portal; last accessed on 6 July 2014). Beginning with the Breast Invasive Carcinoma data set (TCGA, Provisional, *n* = 1037), each patient was classified as expressing high or low levels of YAP/TAZ profile genes and of the HMGA1 or CCNE2 genes. The genes comprising the YAP/TAZ signature are as follows: KRT34; STXBP6; OLR1; THBS1; INHBA; CTGF; SERTAD4; ANKRD1; HSD3B1; ORC1; CENPM; DAW1; ITGB2; IGFBP3; TGM2; ADAMTS1; BDNF; TMEM171; SERPINE2; PTGS2; CCDC18; PLCB4; DEPDC1B; ZBED2; MATN3; CCNA2; TBXA2R; SERPINE1; SLIT2; BCAR4; ZWINT; RAD51; DIAPH3; MCM10; NAV3; SKA1; SHCBP1; RAD51AP1; DDAH1; RIMS2; RRM2; CDC6; PRR16; DAB2; PLK4; ASF1B; KIF14; FMN2; CDC25C; and GINS2. Statistical independence between the different molecular conditions was calculated using Pearson's Chi-squared contingency table tests in the R/Bioconductor environment.

### Survival analysis

Kaplan–Meier survival curves of distant metastasis-free survival (DMFS) of breast cancer patients were classified according to the expression of CCNE2 or HMGA1 using the survival package. To evaluate the correlation between the CCNE2 expression levels and clinical breast cancer data, we also confirmed our analysis using the gene expression-based Outcome for Breast Cancer web tool (GOBO) [54].

For the single regression Cox gene analysis, we used only a subset of the meta-dataset to compare the gene expression data to the DMFS duration. In particular, we used only the Affymetrics HGU133A platform data to avoid the platform type as a confounding variable in the models.

### Protein phosphorylation analysis

The protein phosphorylation data were obtained from the TCGA breast invasive carcinoma dataset via cBioPortal (http://www.cbioportal.org/public-portal/index.do). In particular, using CCNE2 as the gene of interest in the input form, we selected the proteins displaying differential phosphorylation according to the RPPA data.

### Statistical analysis

The data were analyzed via two-tailed Student's *t* tests, and the results were considered significant at a *P* < 0.05. The results are presented as the mean.

## SUPPLEMENTARY FIGURES


