# Supplementary material for: A novel HMGA1-CCNE2-YAP axis regulates breast cancer aggressiveness
Source: Oncotarget. 2015 May 22;6(22):19087–101. doi: 10.18632/oncotarget.4236 (PMC4662477; doi:10.18632/oncotarget.4236)
Supplement: Supplementary file 1 [file oncotarget-06-19087-s001.pdf]

A novel HMGA1-CCNE2-YAP axis regulates breast cancer aggressiveness

Supplementary Material

a

Symbol	Description	ProbeID	Fold Change	P Value	Score
NCAPG	non-SMC condensin I complex, subunit G	218662_s_at	-4.07	2.5E-10	11.28
MLF1IP	MLF1 interacting protein	218883_s_at	-6.64	6.8E-12	9.98
CCNE2	cyclin E2	205034_at	-4.86	1.8E-09	9.81
CENPF	centromere protein F, 350/400kDa (mitosin)	207331_at	-4.14	7.3E-09	8.63
KIF23	kinesin family member 23	204709_s_at	-5.01	2.4E-11	8.57

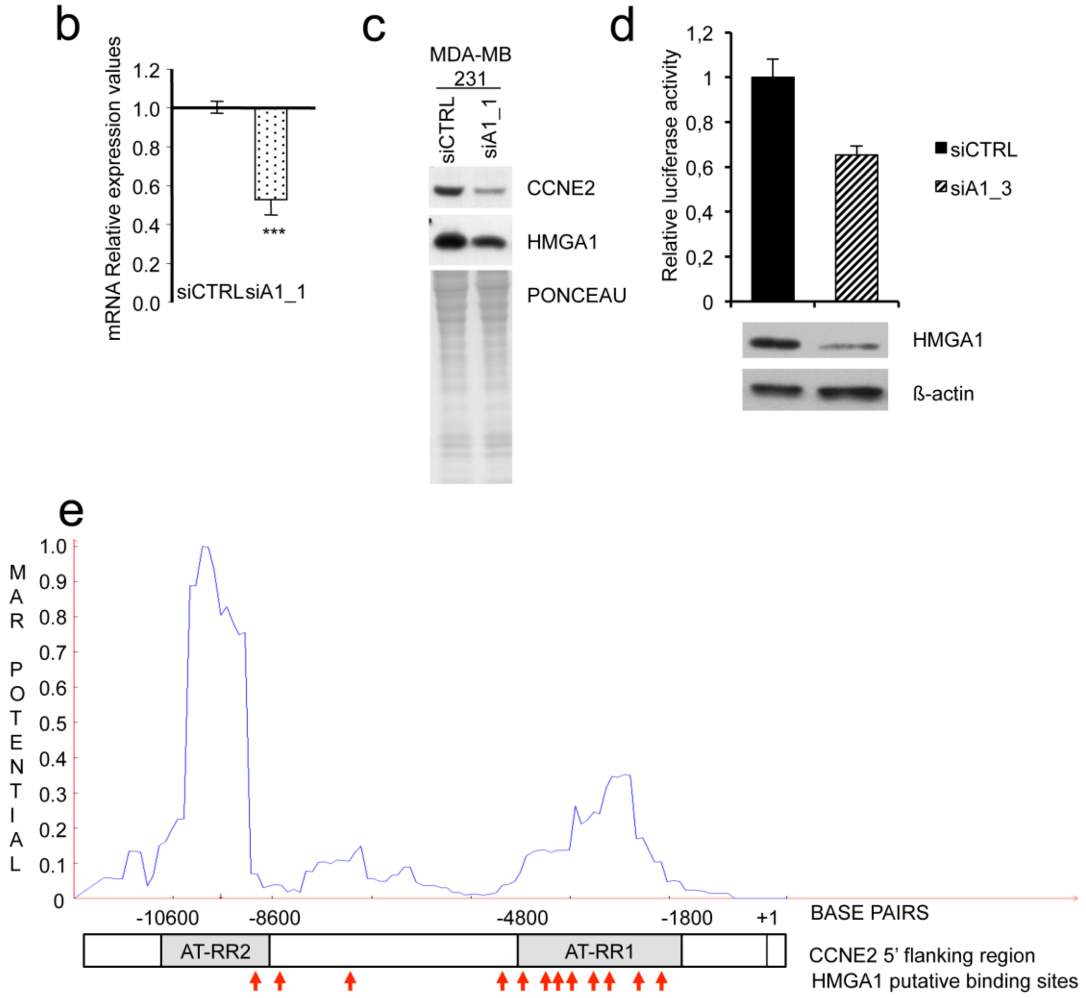

Supplementary Figure S1: (**a**) 130 HMGA gene signature genes were ranked in order to the over all survival. The top five genes are shown. (**b**) mRNA regulation of CCNE2 after HMGA1 silencing (siA1_1) (dotted bar) in MDA-MB-231 was measured using real-time PCR. Expression was compared to the levels in cells transfected with control siRNA (siCTRL). GAPDH was used for

normalization. The data are represented as the means ± SD (n=4). (**c**) Protein regulation of HMGA1 and CCNE2 after HMGA1 silencing (siA1_1) in MDA-MB-231 was presented in a representative western blot. Ponceau coloration was used as loading control. (**d**) HEK293 cells were silenced for HMGA1 and transfected with the luciferase reporter plasmid CCNE2. pRL-CMV Renilla luciferase expression vector was included to normalize for transfection efficiencies. Values are reported as relative luciferase activity in HMGA1 silenced cells compared to control cells (treated with siCTRL). The data are represented as the mean±range between replicates (n≥2). Below the graph Western blot analysis of HMGA1 expression. ß-actin was used as a loading control. (**e**) MAR potential analysis by MAR-Wiz tool of sequence from -11430 to +226 of CCNE2 5'flanking region. Gray boxes correspond to AT-rich regions. Red arrows indicate the HMGA1 putative binding sites calculated with MatInspector tool. ***P<0.001; two-tailed Student's t-test is used throughout.

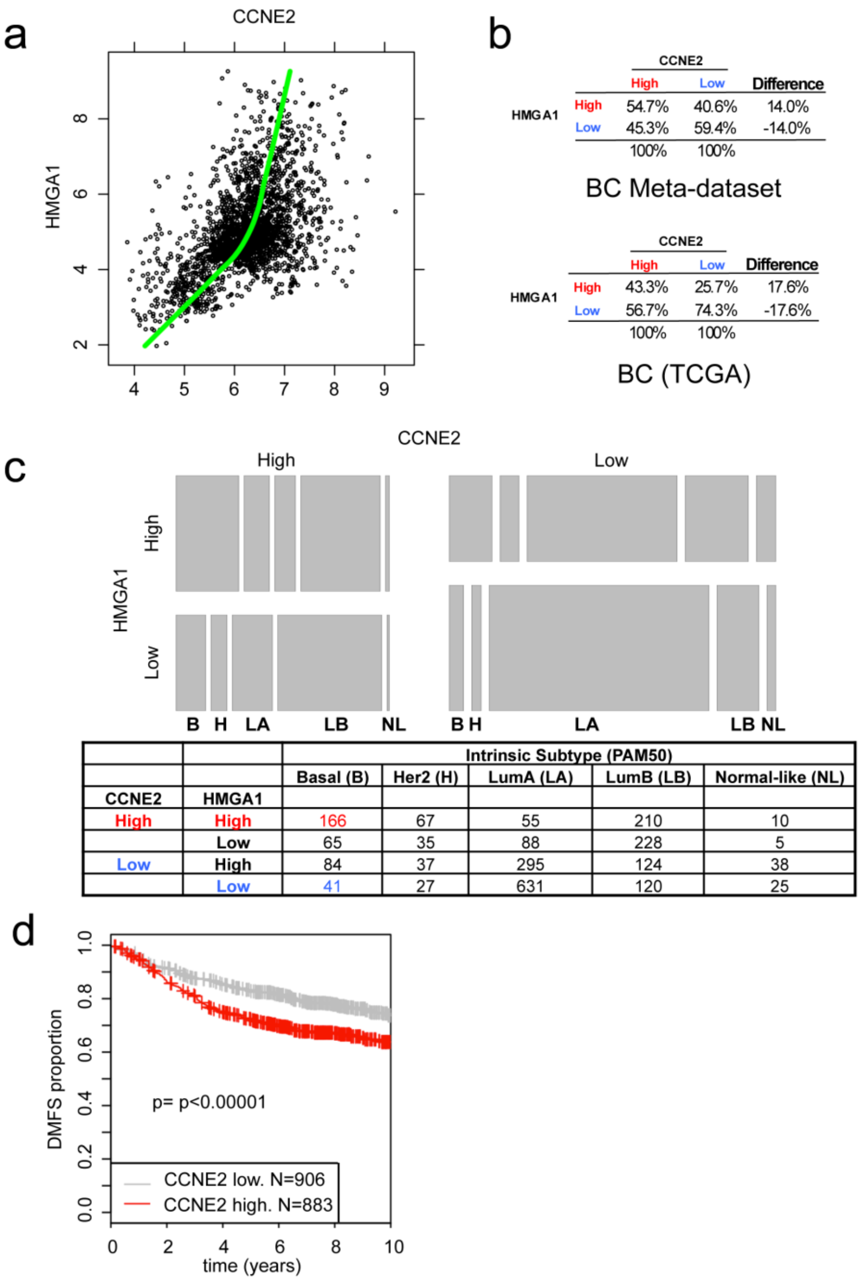

		Intrinsic Subtype (PAM50)				
		Basal (B)	Her2 (H)	LumA (LA)	LumB (LB)	Normal-like (NL)
CCNE2	HMGA1					
High	High	166	67	55	210	10
	Low	65	35	88	228	5
Low	High	84	37	295	124	38
	Low	41	27	631	120	25

Supplementary Figure S2: (**a**) Scatter plot of HMGA1 and CCNE2 expression in the breast cancer meta-dataset. The green line represents the smooth linear regression line ($P<10^{-15}$). (**b**) Contingency table frequencies of breast cancer (BC) samples (meta-dataset and TCGA dataset) classified as having high or low levels of HMGA1 and/or CCNE2. (**c**) Mosaic plot showing the expression levels of the two genes in the breast cancer Meta-dataset. Patients are stratified based on the cancer subtype. (**d**) Kaplan–Meier survival curves showing the relevance of CCNE2 expression for clinical outcome DMFS in breast cancer Meta-dataset.

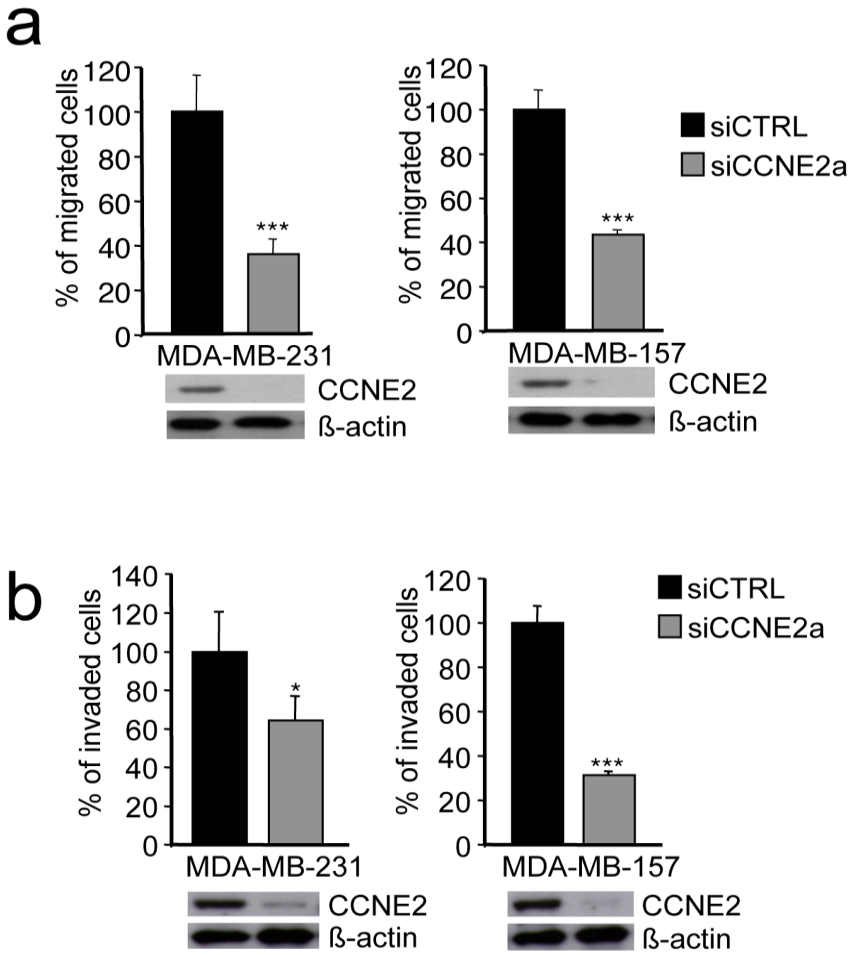

Supplementary Figure S3: (**a**) Transwell migration assay performed on MDA-MB-231 and MDA-MB-157 transfected with siCTRL and siCCNE2a. (**b**) Transwell invasion assay on the same cell lines presented in **a**. Below each graph it is showed a representative western blot of CCNE2 silencing. ß-actin was used as loading control. Data show the means of the percentage of the number of cells relative to control ± SD (n>3). *P<0.05, ***P<0.001; two-tailed Student's t-test is used throughout.

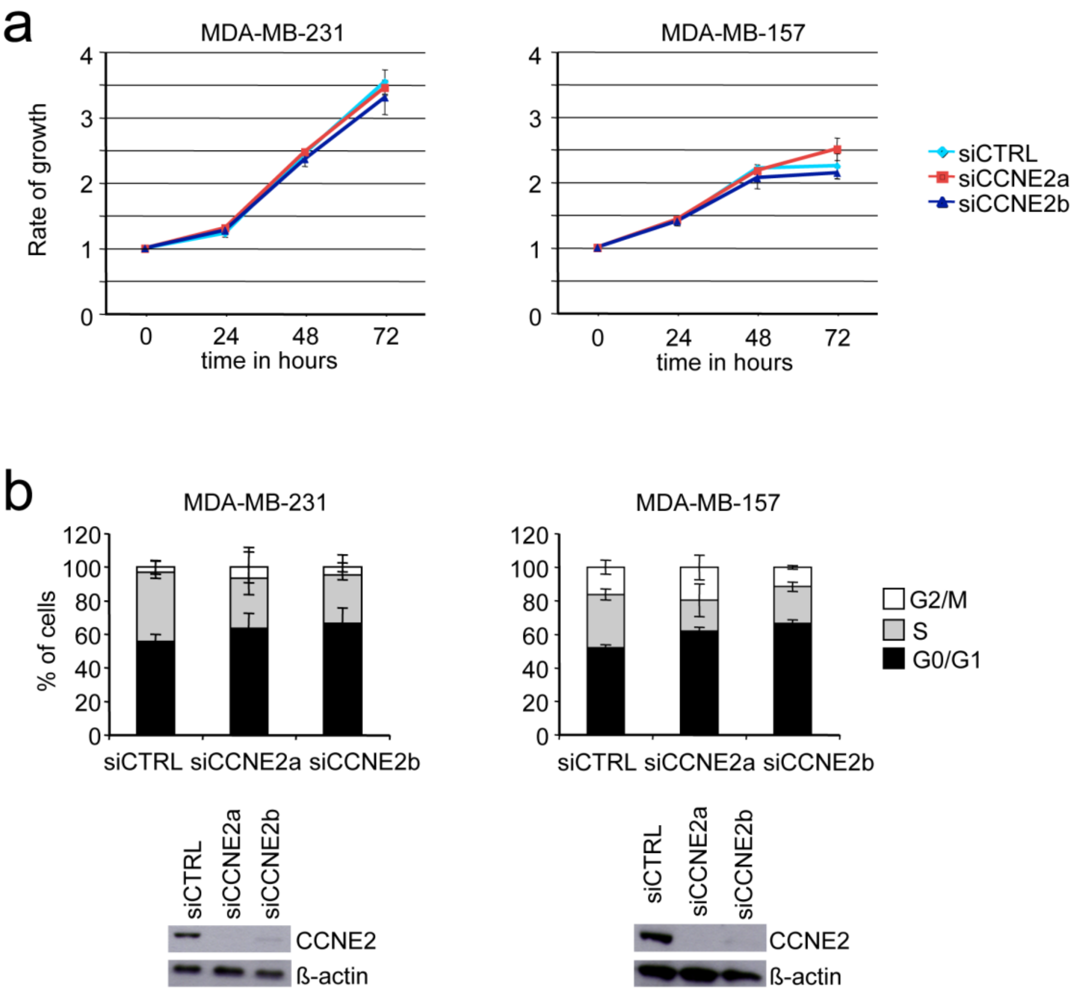

Supplementary Figure S4: (**a**) MTS assay in MDA-MB-231 and MDA-MB-157 silenced for CCNE2 with siCCNE2a and siCCNE2b. Metabolic activity was followed every 24 hours until 72 hours. Data are presented as means ± SD (n≥4). (**b**) Cell cycle was analyzed for DNA content by propidium iodide staining in MDA-MB-231 and MDA-MB-157 after 72 hours from CCNE2 silencing with siCCNE2a and siCCNE2b. Data show the means of the percentage of cells in the different cell cycle phases ± SD (n=5). Below the graph were reported representative western blot of CCNE2 silencing. ß-actin was used as loading control.

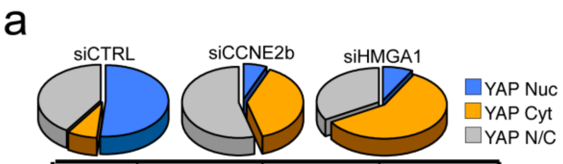

	YAP Nuc	YAP Cyt	YAP N/C
siCTRL	50.8 ± 12.2	7.8 ± 2.7	40.4 ± 10.5
siCCNE2b	6.5 ± 3.3	39.0 ± 3.1	54.5 ± 3.4
siHMGA1	7.8 ± 2.2	59.0 ± 5.6	33.7 ± 4.5

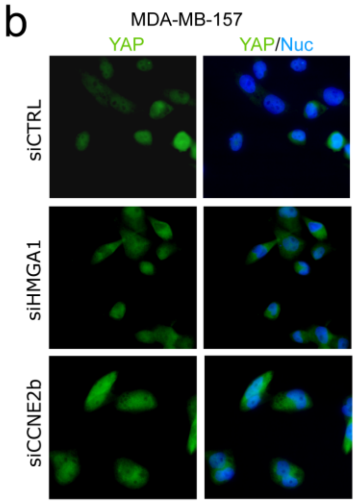

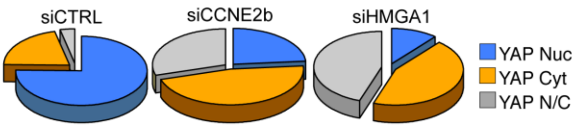

	YAP Nuc	YAP Cyt	YAP N/C
siCTRL	75.23 ± 4.42	21.26 ± 2.01	3.51 ± 3.92
siCCNE2b	23.62 ± 2.04	46.77 ± 4.60	29.61 ± 4.94
siHMGA1	11.01 ± 0.42	44.17 ± 3.69	44.82 ± 3.39

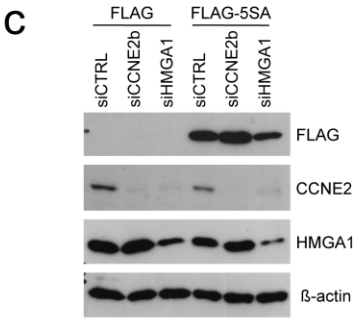

Supplementary Figure S5: (**a**) Quantification of YAP localization in MDA-MB-231 silenced with siCTRL, siCCNE2 and siHMGA1. At least 4 fields for slide (n=3) were counted considering YAP nuclear, cytoplasmic or both localization in cells treated as presented in Figure 4b. (**b**) Immunofluorescence images of YAP in MDA-MB-157 cells after HMGA1 and CCNE2 silencing. Quantification of YAP localization in MDA-MB-157. At least 4 fields for slide (n=3) were counted considering YAP nuclear, cytoplasmic or both localization. (**c**) Representative western blot analysis of data presented in Figure 5c of the cell lysates for FLAG, CCNE2 and HMGA1, using ß-actin as loading control.

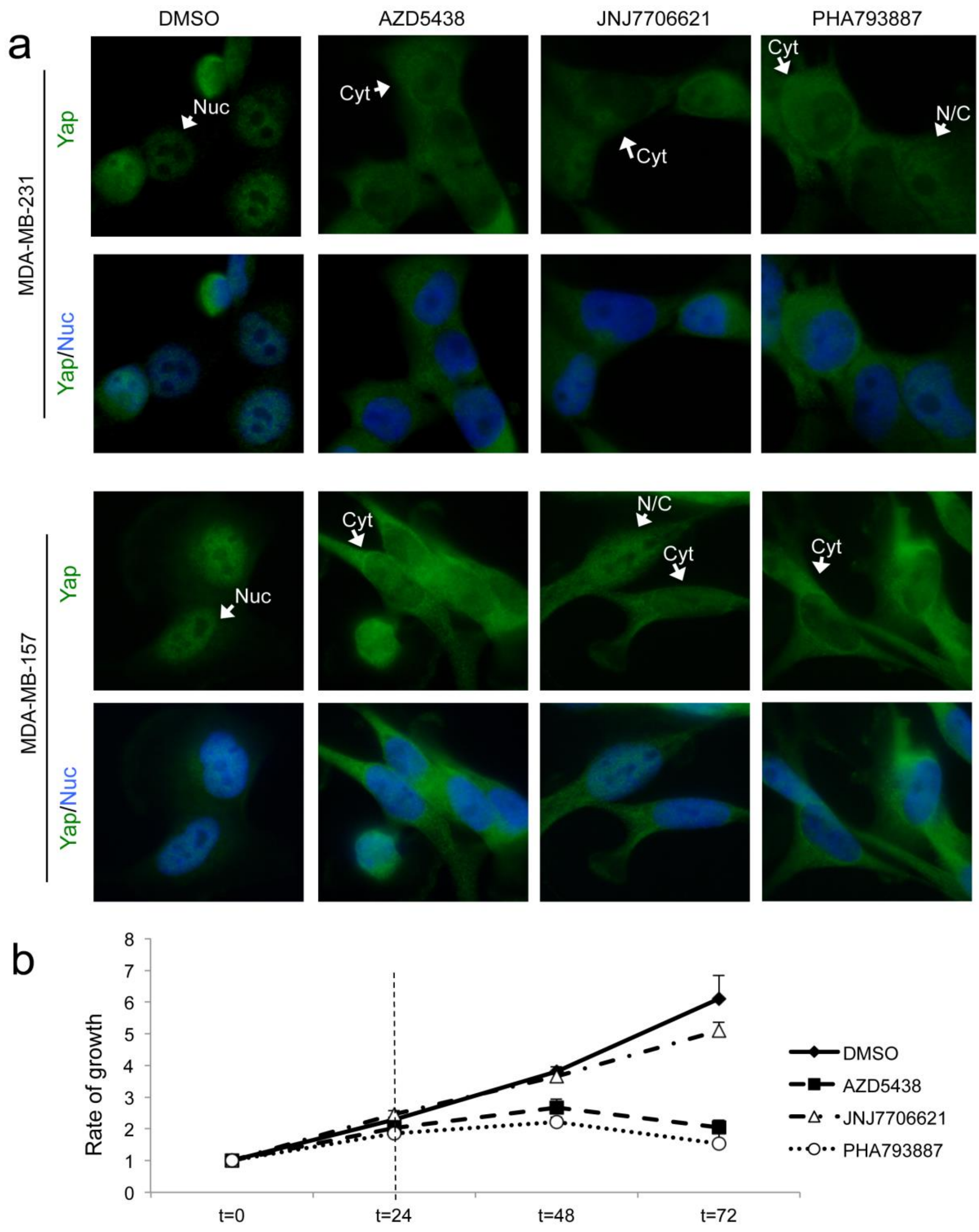

Supplementary Figure S6: (**a**) Immunofluorescence images of YAP in MDA-MB-231 and MDA-MB-157 cells after AZD5438, JNJ7706621 and PHA793887 treatment counted in Figure 6c and 6d respectively. (**b**) MTS assay in MDA-MB-231 treated with DMSO, AZD5438, JNJ7706621 and PHA793887. Metabolic activity was followed every 24 hours until 72 hours. Data are presented as means ± SD (n=5).

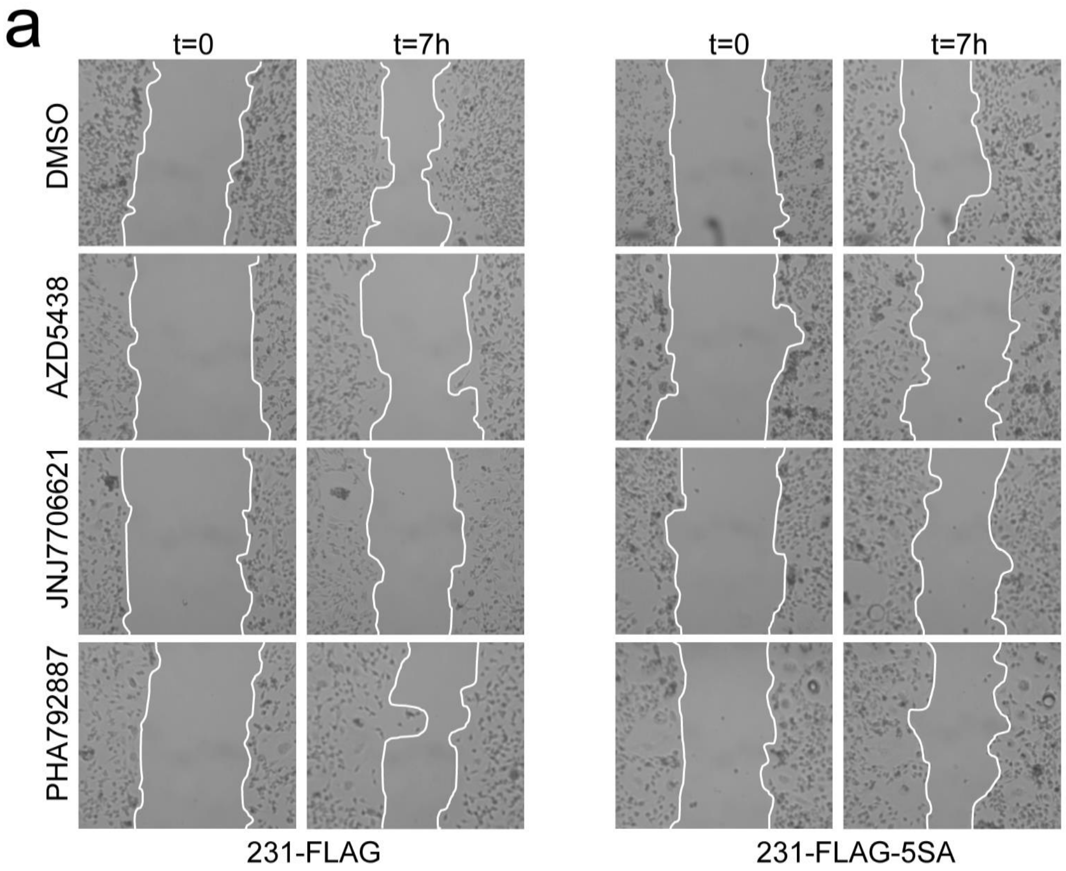

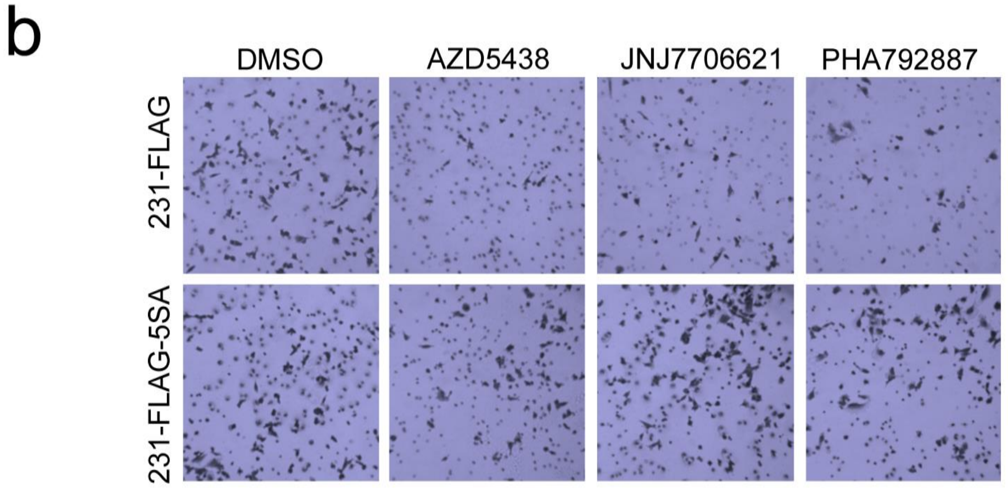

Supplementary Figure S7: (**a**) Representative images of wound healing assay at the time considered in the analysis presented in Figure 6g. (**b**) Representative images of transwell assay at the time considered in the analysis presented in Figure 6h.
